# Biofluid Specificity of Long Non-Coding RNA Profile in Hypertension: Relevance of Exosomal Fraction

**DOI:** 10.3390/ijms23095199

**Published:** 2022-05-06

**Authors:** Angela L. Riffo-Campos, Javier Perez-Hernandez, Olga Martinez-Arroyo, Ana Ortega, Ana Flores-Chova, Josep Redon, Raquel Cortes

**Affiliations:** 1 Millennium Nucleus on Sociomedicine (SocioMed) and Vicerrectoría Académica, Universidad de La Frontera, Temuco 4780000, Chile; angela.riffo@ufrontera.cl; 2 Department of Computer Science, ETSE, University of Valencia, 46010 Valencia, Spain; 3 Cardiometabolic and Renal Risk Research Group, INCLIVA Biomedical Research Institute, 46010 Valencia, Spain; javier.perez-hernandez@inserm.fr (J.P.-H.); omartinez@incliva.es (O.M.-A.); afloreschova@gmail.com (A.F.-C.); josep.redon@uv.es (J.R.); 4 Department of Nutrition and Health, Valencian International University (VIU), 46002 Valencia, Spain; 5 T-Cell Tolerance, Biomarkers and Therapies in Type 1 Diabetes Team, Institut Cochin CNRS, INSERM, Université de Paris Cité, F-75014 Paris, France; 6 Internal Medicine Unit, Hospital Clinico Universitario, 46010 Valencia, Spain; 7 CIBER of Physiopathology of Obesity and Nutrition (CIBEROBN), Institute of Health Carlos III, Minister of Health, 28029 Madrid, Spain

**Keywords:** hypertension, urinary albumin excretion, long non-coding RNA, exosomes

## Abstract

Non-coding RNA (ncRNA)-mediated targeting of various genes regulates the molecular mechanisms of the pathogenesis of hypertension (HTN). However, very few circulating long ncRNAs (lncRNAs) have been reported to be altered in essential HTN. The aim of our study was to identify a lncRNA profile in plasma and plasma exosomes associated with urinary albumin excretion in HTN by next-generation sequencing and to assess biological functions enriched in response to albuminuria using GO and KEGG analysis. Plasma exosomes showed higher diversity and fold change of lncRNAs than plasma, and low transcript overlapping was found between the two biofluids. Enrichment analysis identified different biological pathways regulated in plasma or exosome fraction, which were implicated in fatty acid metabolism, extracellular matrix, and mechanisms of sorting ncRNAs into exosomes, while plasma pathways were implicated in genome reorganization, interference with RNA polymerase, and as scaffolds for assembling transcriptional regulators. Our study found a biofluid specific lncRNA profile associated with albuminuria, with higher diversity in exosomal fraction, which identifies several potential targets that may be utilized to study mechanisms of albuminuria and cardiovascular damage.

## 1. Introduction

Hypertension (HTN) has been declared a worldwide epidemic; besides posing a substantial risk factor for cardiovascular diseases, it is also a leading cause of chronic kidney disease [1]. Both genetic and environmental factors contribute to disease development, although their relative contributions are still poorly defined. Epigenetic phenomena such as histone modification, DNA methylation, chromatin remodelling, and especially, non-coding RNA (ncRNA)-mediated targeting of various genes regulate molecular mechanisms involved in the pathogenesis of HTN [2].

Based on the size, ncRNAs are classified as long (>200 nucleotides), intermediate (~20–200 nucleotides), or small (~20 nucleotides). Small ncRNAs include small interfering RNAs (siRNAs), piwi-interacting RNAs (piRNA), and microRNAs (miRNAs) [3]. Intermediate ncRNAs include transfer RNA (tRNA), small nuclear ribonucleic acid RNA (snRNA), and nucleolar RNAs (snoRNA,) which modify ribosome RNA [3]. The ncRNAs greater than 200 nucleotides have been grouped into long ncRNAs (lncRNAs). LncRNAs have numerous functions in regulating gene expression with chromatin imprinting, splicing, transcription and translation, and cell cycle development [4]. lncRNAs can silence multiple genes through recruiting transcriptional factors to target promoters and induce transcription or by interacting with chromatin; thus, they are essential to the epigenetic control of gene expression [5].

Research on the relationship between ncRNAs and cardiovascular diseases, especially HTN, has been increasing in recent years [6,7]. In addition, bioinformatics can provide clues for determining the downstream targets or binding proteins of ncRNA and how these ncRNAs work [8,9]. In a previous study, we constructed a lncRNA–miRNA–mRNA regulatory network with the ncRNA signature, combining bioinformatics and correlation analyses associated with the development of urinary albumin excretion (UAE) [10]. However, except in HTN-associated diseases such as coronary artery disease [11], pulmonary arterial HTN [12,13], or preeclampsia [14], scarce circulating lncRNAs were reported to be altered in essential HTN [15,16,17].

ncRNAs can be transported into extracellular vesicles (EVs), mainly exosomes, and may act as paracrine effectors in the cross talk between several cell types [18]. Previous studies have established exosomal ncRNAs as biomarkers in HTN [19,20]. Our group has recently found that plasma and urinary-derived exosomes have a different miRNA signature related to albuminuria in HTN, reflecting changes that occur in the kidneys [21]. Nevertheless, the study of the lncRNA profiling in circulating plasma and exosomes associated with early renal damage in HTN remains mainly unknown.

The aim of this study was to identify the lncRNA profiles in circulating plasma and packaged them into exosomes associated with UAE in HTN using deep sequencing technology. We also assessed the effect of the biofluid origin on lncRNA signature and regulated pathways.

## 2. Results

### 2.1. Study Population

The study cohort included 48 essential hypertensive subjects, 26 normoalbuminurics (Non-UAE) and 22 with increased UAE. General patient characteristics are shown in Table 1.

### 2.2. Non-Coding RNA Biotype Distribution According to Biofluid, Plasma, and Plasma Exosomes

**Figure 1:**
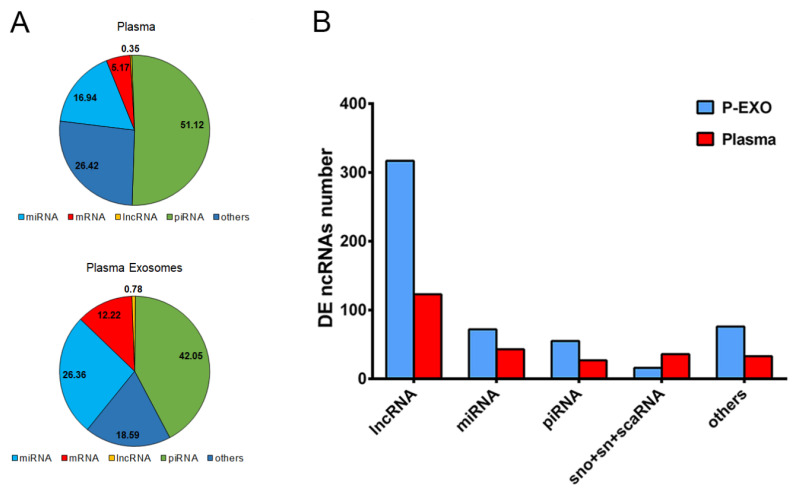
Proportions of RNA types in each biological fraction. (**A**) Representation of RNA biotype mapped reads in each biological fluid from hypertensive patients. The unit used was filtered, normalized, and counts per million (CPM) mapped reads annotated. (**B**) Characterization of differentially expressed (DE) non-coding RNA type in hypertensive patients in plasma exosome (P-EXO) (blue) and plasma (red). miRNA: microRNA; mRNA: messenger RNA; piRNA: PIWI-interacting RNA; rRNA: ribosomal RNA; snoRNA: small nucleolar RNA; snRNA: small nuclear RNA; scaRNA: small cajal body RNA.

Detection of RNA biotypes according to biofluid was performed using Small-RNA-sequencing single-end technology comparing two biofluids (plasma and plasma exosomes). When we analyzed the mapped reads comparing biofluid types, we observed that piRNAs and miRNAs are the most representative RNA biotypes in both plasma and plasma exosomes, followed by others, including pseudogenes, Y-RNA, Vault-RNA, small nucleolar RNA (snoRNA), small nuclear RNA (snRNA) and small cajal body RNA (scaRNA), mRNA, and lncRNA (Figure 1A). Mapped reads in the piRNA group were more augmented in plasma than in plasma exosomes (51.1% and 42.0%, respectively), and the miRNA group was more augmented in plasma exosomes than in plasma (26.36% and 16.94%, respectively). We next sought to analyze the proportion of RNA biotypes present in each of the two biological biofluids comparing patient groups with and without UAE; here, nonsignificant differences were observed (Supplementary Figure S1).

Then, when we analyzed the differentially expressed (DE) ncRNA among hypertensive patients with or without UAE, we observed that lncRNA was the predominant ncRNA biotype with statistically significant transcripts in both plasma and plasma exosomes, representing the 54% of total transcripts, followed by miRNAs (15%) and piRNAs (10%). When we compared these data according to biofluid origin, we could observe that plasma exosomes always showed a higher number of DE ncRNA transcripts than plasma, above all in lncRNA and miRNA (317 transcripts compared with 123, respectively), except for the snoRNA and snRNA groups that were augmented in plasma compared with an exosomal fraction (36 vs. 16) (Figure 1B). However, when comparing the RNA biotype percentages of total transcripts, no significant differences were found, except for the lncRNA group (58% in plasma exosomes and 47% in plasma).

These data indicate that biofluid origin influences RNA type distribution, mainly in the predominant ncRNA biotypes, plasma exosomes being an ncRNA/lncRNA-enriched fraction.

### 2.3. Long Non-Coding RNA Biotype Overlapping between Biofluids

**Figure 2:**
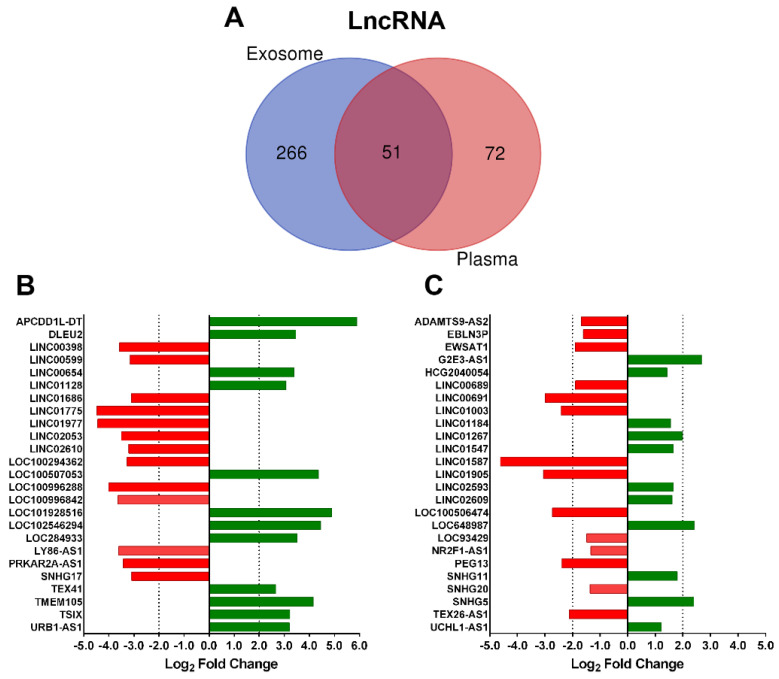
Differentially expressed lncRNA in each biological fluid. (**A**) Venn diagram shows the overlap among biological fractions. (**B**) Top 25 lncRNA with the highest *p*-value and log2 FC ≥ 2 or ≤−2 in plasma exosomes. (**C**) Top 25 lncRNA with the highest *p*-value and log2 FC ≥ 2 or ≤−2 in plasma. Downregulated lncRNAs are in red, and upregulated lncRNAs are in green.

Next, we analyzed the influence of biofluid specificity on DE ncRNA in the most representative biotypes. Focusing on the lncRNA group, the Venn diagram obtained from the biological compartments showed very limited overlapping between exosomal and plasma fractions; 84% was specific to plasma exosomes and 59% to plasma fraction (Figure 2A). The majority of unique DE lncRNA in the exosome fraction were downregulated (71%) in albuminuric patients and 61% in plasma samples. For the other three groups, miRNA and piRNA, the exosomal fraction showed the highest specificity; however, for sno+snRNA, only 45% was specific to plasma exosome and 69% to plasma (Supplementary Figure S2).

In addition, diverging bar charts for lncRNA biotype showed the fold change (FC) expression of the top 25 unique transcripts in exosome-enriched fraction and plasma (Figure 2B,C). In both groups, approximately 50% of lncRNAs were significantly downregulated in albuminuric patients, and plasma lncRNA showed lower FC; only 40% of statistically significant transcripts had a log_2_ FC ≥ 2 or ≤−2, compared with 100% top lncRNAs in exosomal fraction (Supplementary Table S1).

### 2.4. Functional Enrichment Analysis of the Differentially Expressed lncRNAs from Hypertensive Patients with Urinary Albumin Excretion

**Figure 3:**
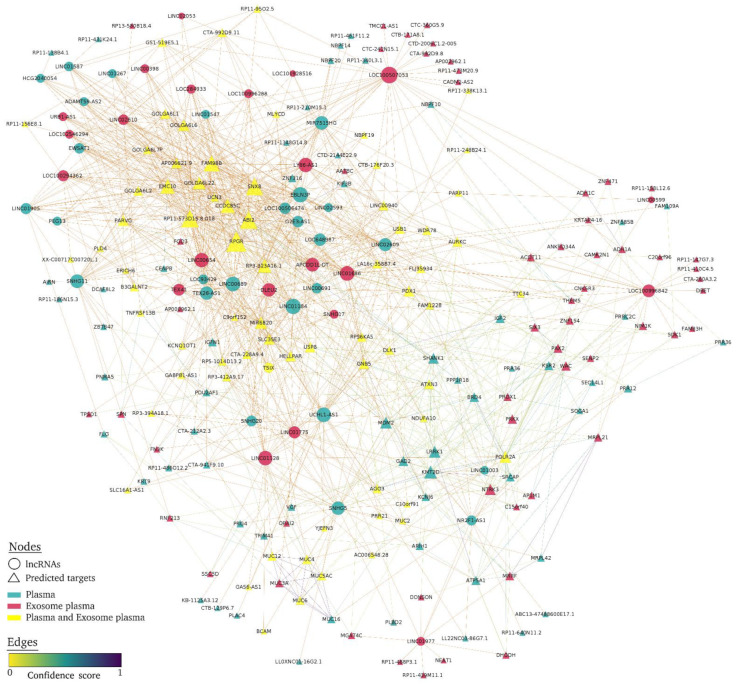
Overview of lncRNA-target interaction network related to HTN-associated UAE. Each lncRNA in the two biofluids is a circle node, lncRNA targets are triangle nodes, node size increases according to the number of edges (network degree), and a higher confidence score indicates a stronger edge between nodes. The plasma fraction is in green, plasma exosome is in red, and common plasma and plasma exosomes are in yellow.

**Figure 4:**
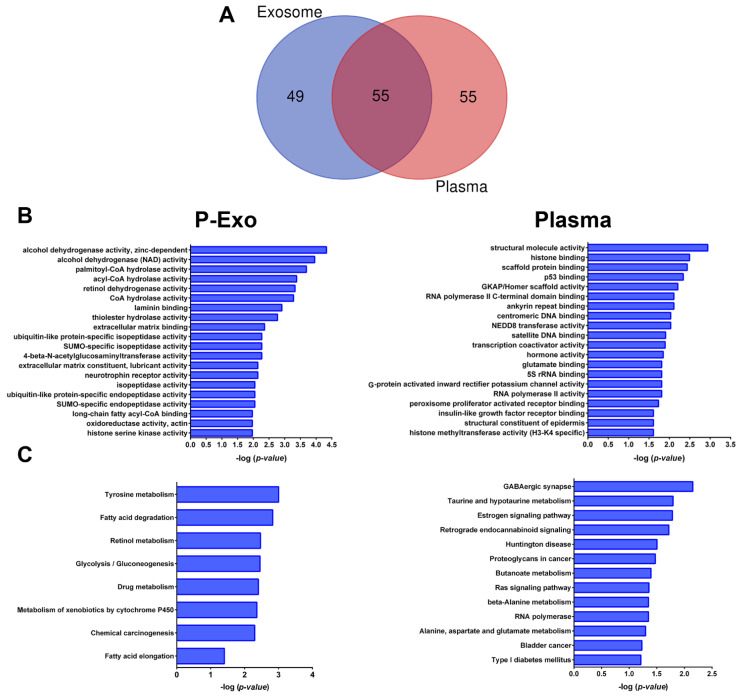
Function enrichment analyses of long ncRNA targets. (**A**) Venn diagram shows the lncRNA targets overlapping among biological fractions. (**B**) The top 20 most significant Gene Ontology terms for plasma exosome and plasma, respectively. (**C**), The top 20 most significant Kyoto Encyclopedia of Genes and Genomes (KEGG) pathway terms for plasma exosomes and plasma, of the top 25 unique lncRNAs for each biofluid. P-Exo—plasma exosomes.

To focus on the lncRNAs strongly associated with albuminuria, the top 25 DE lncRNAs in both exosome and plasma fractions were selected, potential predicted targets were identified (Supplementary Table S2), and the lncRNA targets network was constructed (Figure 3). For plasma, 26.4% of targets were lncRNAs and one miRNA (miR-6820) from the total, and for exosome fraction, 27.8% were lncRNA and one miRNA (miR-6820). LncRNA targets network showed that the common predicted lncRNA targets were those that had a greater number of interactions between nodes (larger triangle size), and several exosomal lncRNA (LOC100507053, LOC100996842, and LINC01977) mainly targeted other lncRNAs (Figure 3). Supplementary Table S3 shows the targets with the highest network degree (greater than 10), betweenness, and closeness centrality. In addition, the Venn diagram obtained from among the biological compartments showed high overlapping between lncRNA targets, of 50% in plasma and 52.8% for plasma exosome fraction (Figure 4A).

Finally, to further understand the biological functions of the unique lncRNA targets (n = 49 in plasma exosome and n = 55 in plasma), we performed the over-representation analysis (ORA) using GO functional annotations and KEGG pathway analysis for each biofluid. Functional enrichment analysis uncovered structural molecule activity, histone binding, scaffold protein binding and p53 binding for plasma; and acyl CoA hydrolase, laminin binding, extracellular matrix binding, and ubiquitin-like protein-specific isopeptidase activity, among other GO terms associated with plasma exosomes (Figure 4B). The ORA further revealed 13 enriched KEEG terms for plasma and 8 enriched KEGG terms for plasma exosome (Figure 4C). The involved transcripts in the most relevant pathways identified are listed in Supplementary Table S4, and their biological implications are discussed in the next section.

## 3. Discussion

The present study identified a lncRNA profile specific to biofluid, circulating in plasma or packaged into exosomes, associated with UAE in HTN. Low DE lncRNA overlapping was observed between the two biofluids, the exosome-enriched fraction showing higher lncRNA diversity and higher fold change than plasma. In addition, we used Gene Ontology (GO) and Kyoto Encyclopedia of Genes and Genomes (KEGG) pathway analysis to assess the enriched biological functions regulated by the lncRNA signatures, showing a specific enrichment according to each biofluid. Exosomal lncRNAs were implicated mainly in mechanisms regulating fatty acid metabolism, extracellular matrix (ECM) regulation and remodelling, and sorting of non-coding RNAs into exosomes, while plasma lncRNAs were involved in genome reorganization, interference with RNA polymerase machinery, and as scaffolds for assembling transcriptional regulators.

Research on the relationship between lncRNA and cardiovascular diseases, especially HTN, is limited [2]. Experimental approaches with cell and animal models have identified diverse tissue and cellular lncRNAs associated with endothelial function and vascular remodelling [22,23]. However, some circulating lncRNAs have been shown to play a role in the pathophysiological regulation of blood pressure [15,16,17,24].

The most striking finding of this study is that using next-generation sequencing to screen for a specific lncRNA profile and the use of two biofluids (plasma and plasma exosomes) has allowed us to establish a biofluid-specific lncRNA signature associated with albuminuria in HTN. We observed that the DE lncRNA levels circulating in plasma differed from their levels when packaged into exosomes; a greater percentage of significant transcripts were found in the exosomal fraction rather than in plasma. Compared with our previous work, in which we selected common ncRNA in three biofluids [10], in this current manuscript, specific lncRNA from exosomal fraction showed the highest fold changes and most of the transcripts were upregulated. In addition, we found low lncRNA overlapping between the two biofluids, pointing to a controlled and specific process related to a pathological condition such as HTN. This is consistent with previous studies, which found selective sorting of specific ncRNAs into exosomes compared with the whole ncRNA circulating pool in specific biological or disease conditions [25,26].

Another strength of our study is the GO and KEGG pathway analyses assessing biological functions enriched in response to albuminuria in plasma and plasma exosomes, which has allowed us to support whether, besides lncRNA profile differences, there were also differences in the pathways that regulate plasma or exosome-specific lncRNAs. In the plasma fraction, the enrichment analysis highlighted several pathways associated with histone or chromatin remodelling (histone binding, centromeric and satellite DNA binding, histone methyltransferase activity, etc.), RNA polymerase, and scaffold protein binding. In plasma exosome-enriched fraction, the highlighted pathways were associated with lipid metabolism (CoA hydrolase activity, thiolester hydrolase), ECM (laminin binding and basal cell adhesion), and with mechanisms of sorting ncRNAs into exosomes (SUMOylation, a ubiquitin-like protein, etc.).

LncRNAs have highly diverse functions, ranging from regulating gene expression at transcriptional and epigenetic levels in the nucleus to controlling mRNA turnover and translation in the cytosol [27]. Accumulating evidence points to a regulatory role for lncRNA in genome organization by modulating chromatin compaction and establishing chromosomal contacts [28]. Our results showed that the plasma lncRNA profile regulated pathways implicated in the histone binding, centromeric, and satellite DNA binding by controlling bromodomain-containing protein 4 (BRD4) and DNA binding of centromere protein (CNEP) genes, respectively [29,30]. BRD4, an epigenetic reader, is one of the most important BET proteins and plays an important role in angiogenesis and the development of inflammation-associated and cardiovascular diseases, central nervous system disorders and cancers [29]. In addition to indirect effects on gene expression, lncRNAs can directly regulate transcription by forming R-loops, interfering with RNA polymerase machinery, and transcription of the lncRNA locus [31]. Our lncRNA profile regulates several targets implicated in RNA polymerase II C-terminal domain (BRD4, POLR2A), serving as a flexible binding scaffold for numerous nuclear factors; which factor binds is determined by the phosphorylation patterns on the CTD repeats [32]. An emerging concept is that the association between certain lncRNAs with the disease may involve their scaffolding ability, forming ribonucleoproteins and bringing proteins into proximity [33]. Our plasma lncRNA profile includes several scaffold pathways, GKAP/Homer scaffold activity [34], and 5S rRNA binding through MDM2 [35]. Previous work by systems biology analysis revealed an important functional role for MDM2 in glomeruli and tubules of the diabetic nephropathic kidney [36].

The plasma exosome lncRNA profile identified targeted biological pathways implicated in fatty acid metabolism, such as CoA hydrolase activity, thiolester hydrolase activity, and acyl-CoA binding. Exosomes exert remarkable effects on lipid metabolism, including lipid synthesis, transportation, and degradation, and more importantly, lipid metabolism can also affect exosome production and secretion [37]. Acyl-CoA products regulate metabolic enzymes and signalling pathways and are incorporated into acylated proteins and complex lipids such as triacylglycerol, phospholipids, and cholesterol esters [38]. Phospholipids, which are components of the EV membrane, play important roles in cell intercommunication and lipid mediators generation [39]. Thus, ncRNAs and lipids could mediate the EVs function. Another regulated biological pathway is related to ECM binding and laminin binding through the BCAM receptor and SSC5 [40]. Previous studies have established that lncRNAs could act as a novel regulatory system for modulation of ECM conformation and, therefore, the phenotype of the cells located in this environment [41,42]. Finally, an important biological pathway related to our exosomal lncRNA profile was associated with mechanisms for sorting protein and ncRNAs into exosomes (SUMOylation and ubiquitin-like proteins) [43]. SUMO-specific proteases (SENPs) have a key role in human desumoylation, and recent work showed that plasma exosome-derived SENPs have potential prognostic value [44].

In summary, our study revealed a distinct lncRNA profile circulating in plasma and packaged into exosomes associated with albuminuria. This regulates different biological pathways involved in transcriptional and post-transcriptional mRNA regulation according to biofluid and is ultimately related to pathological HTN. Experimental validation of our findings is necessary to establish their potential as diagnostic and therapeutic targets for albuminuria development in HTN.

## 4. Materials and Methods

### 4.1. Subjects and Samples

This was an observational case–control study which included 26 patients with essential HTN without UAE (≥30 mg/g urinary creatinine) and 21 patients with persistent elevated UAE [45]. Hypertensive patients with uncontrolled HTN, resistant HTN, secondary HTN, or severe kidney disease were excluded. The samples correspond to small RNA-Seq raw data from two biofluid fractions (plasma-derived exosomes and total plasma).

Human blood samples were processed within one hour after reception to isolate the exosomal fraction. Exosomes were isolated from plasma (P-Exo) using sequential ultracentrifugation, and pellets were characterized by transmission electron microscopy, Western blot and qNano Gold instrument (Izon Science Ltd., Christchurch, New Zealand). Detailed protocols are explained in our previous study [21].

### 4.2. RNA Extraction, Small RNA Library Preparation, and Next-Generation Sequencing

Total RNA was extracted from plasma samples using the miRNeasy mini kit (Qiagen, Hilden, Germany) and from exosomes using the Total Exosome RNA and Protein Isolation kit (Invitrogen, Life Technologies, Carlsbad, CA, USA). Quantification of total RNA, size, and quality distribution were analyzed by capillary electrophoresis (Agilent 2100 Bioanalyzer, Agilent Technologies, Santa Clara, CA, USA). Individual patient libraries were performed using 2 µL of total RNA from each biofluid (P-Exo or plasma) using the CleanTag Small RNA library preparation kit (TriLink Biotechnologies, San Diego, CA, USA), as previously described [46]. Pools were sequenced on the HiSeq 2000 platform (Illumina, San Diego, CA, USA) with a 50-cycle single-read mode (CNAG, Barcelona, Spain). The raw RNA-Seq dataset is included in the BioProject repository, accession PRJNA590749.

### 4.3. Small RNA Sequencing Data Analysis

The detailed pipeline analysis followed was explained in our previous article [10]. In summary, raw data were quality controlled by FastQC v0.11.8 [47] and were filtered using FASTX-Toolkit v0.013, and alignment was made with STAR v2.7.3a [48]. GENCODE human genome release 38 (GRCh38.p13) was used as a reference genome. SAM files were converted to BAM and sorted with SAMtools v1.10 [49]. The count matrix was obtained using GenomicFeatures, Rsamtools, and GenomicAlignments Bioconductor packages [50,51]. Then, the count matrix was included in a DGEList object using the edgeR Bioconductor package [52], annotation was performed, and a matrix with filtered, normalized, and annotated counts per million (CPM) mapped reads was generated to estimate the abundance of RNA types in each group.

### 4.4. Statistical Analysis

Comparisons between hypertensive patients without albuminuria and with UAE were performed by a negative binomial generalized log-linear model, adjusted for sex. The *p*-values were adjusted using Benjamini–Hochberg method, and *p* < 0.05 was considered statistically significant. The R ggplot2 or VennDiagram packages were used for making graphs. Statistical analyses were performed using the edgeR Bioconductor package [52].

### 4.5. Long Non-Coding RNA Target Predictions and Molecular Pathways Analyses

LncRNA targets were predicted using LncRRIsearch, a web server for lncRNA–lncRNA and lncRNA–mRNA interaction (http://rtools.cbrc.jp/LncRRIsearch, accessed on 13 February 2022) [53]. The top ten targets with an energy threshold <−100 kcal/mol for each isoform were selected. Then, the web-based Gene SeT AnaLysis Toolkit (http://www.webgestalt.org/, accessed on 15 February 2022) was used to Gene set ORA through GO terms and KEGG pathways [54]. The ncRNA target network was generated using STRING database v11.0 [55], selecting biological interactions with a confidence score of ≥0.2. Then, the networks were analyzed and displayed with Cytoscape v3.8.1 [56]; nodes and edges represented biological data, with edges representing interactions between nodes and each node representing a biological molecule.

## Figures and Tables

**Table 1:** Clinical characteristics of the study cohort.

Variables	Albuminuria (UAE) (n = 22)	Normoalbuminuria (Non UAE) (n = 26)
Age (years)	52.2 ± 8.3	55.0 ± 5.3
Gender (male)	68.2%	65.4%
SBP (mmHg)	136 ± 15	136 ± 24
DBP (mmHg)	85 ± 10	87 ± 14
Glucose (mg/dL)	122 ± 46	119 ± 41
Total Cholesterol (mg/dL)	200 ± 34 **	173 ± 29
LDL (mg/dL)	128 ± 30 **	108 ± 25
HDL (mg/dL)	51 ± 14	50 ± 10
Triglycerides (mg/dL)	153 ± 78	127 ± 60
Plasma creatinine (mg/dL)	0.87 ± 0.30	0.90 ± 0.22
GFR (mL/min/1.73 m^2^)	96 ± 27	87 ± 19
Body mass index (kg/m^2^)	32 ± 7	30 ± 5
Obesity grade (%) Grade I Grade II Grade III	29 9 14	20 12 8
Diabetes (%)	41	35
Dyslipidemia (%)	86	85
Smoking (%)	55	48
UAE/Creatinine (mg/g)	146.4 ± 144.3 ***	3.1 ± 1.7

DBP—diastolic blood pressure; GFR—glomerular filtration rate; HDL—high density lipoprotein; LDL—low density lipoprotein; SBP—systolic blood pressure; UAE—urinary albumin excretion. ** *p* value < 0.001; *** *p* value < 0.0001.

## Data Availability

The raw RNA-Seq dataset is available at the BioProject repository, accession: PRJNA590749.

## Supplementary Materials

The following supporting information can be downloaded at: https://www.mdpi.com/article/10.3390/ijms23095199/s1.

## References

[1] Williams B., Mancia G., Spiering W., Agabiti Rosei E., Azizi M., Burnier M., Clement D.L., Coca A., De Simone G., Dominiczak A. (2018). 2018 ESC/ESH Guidelines for the management of arterial hypertension: The Task Force for the management of arterial hypertension of the European Society of Cardiology (ESC) and the European Society of Hypertension (ESH). J. Hypertens..

[2] Wu G., Jose P.A., Zeng C. (2018). Noncoding RNAs in the Regulatory Network of Hypertension. Hypertension.

[3] Beermann J., Piccoli M.-T., Viereck J., Thum T. (2016). Non-coding RNAs in Development and Disease: Background, Mechanisms, and Therapeutic Approaches. Physiol. Rev..

[4] Asl M.H., Khelejani F.P., Mahdavi S.Z.B., Emrahi L., Jebelli A., Mokhtarzadeh A. (2022). The various regulatory functions of long noncoding RNAs in apoptosis, cell cycle, and cellular senescence. J. Cell. Biochem..

[5] Yao R.-W., Wang Y., Chen L.-L. (2019). Cellular functions of long noncoding RNAs. Nat. Cell Biol..

[6] Chen S., Chen R., Zhang T., Lin S., Chen Z., Zhao B., Li H., Wu S. (2018). Relationship of cardiovascular disease risk factors and noncoding RNAs with hypertension: A case-control study. BMC Cardiovasc. Disord..

[7] Matshazi D.M., Weale C.J., Erasmus R.T., Kengne A.P., Davids S.F.G., Raghubeer S., Davison G.M., Matsha T.E. (2021). Circulating Levels of MicroRNAs Associated With Hypertension: A Cross-Sectional Study in Male and Female South African Participants. Front. Genet..

[8] Chen K., Ma Y., Wu S., Zhuang Y., Liu X., Lv L., Zhang G. (2019). Construction and analysis of a lncRNA-miRNA-mRNA network based on competitive endogenous RNA reveals functional lncRNAs in diabetic cardiomyopathy. Mol. Med. Rep..

[9] Zou J.-B., Chai H.-B., Zhang X.-F., Guo D.-Y., Tai J., Wang Y., Liang Y.-L., Wang F., Cheng J.-X., Wang J. (2019). Reconstruction of the lncRNA-miRNA-mRNA network based on competitive endogenous RNA reveal functional lncRNAs in Cerebral Infarction. Sci. Rep..

[10] Riffo-Campos A.L., Perez-Hernandez J., Ortega A., Martinez-Arroyo O., Flores-Chova A., Redon J., Cortes R. (2022). Exosomal and Plasma Non-Coding RNA Signature Associated with Urinary Albumin Excretion in Hypertension. Int. J. Mol. Sci..

[11] Elwazir M.Y., Hussein M.H., Toraih E.A., Al Ageeli E., Esmaeel S.E., Fawzy M.S., Faisal S. (2022). Association of Angio-LncRNAs MIAT rs1061540/MALAT1 rs3200401 Molecular Variants with Gensini Score in Coronary Artery Disease Patients Undergoing Angiography. Biomolecules.

[12] Omura J., Habbout K., Shimauchi T., Wu W.-H., Breuils-Bonnet S., Tremblay E., Martineau S., Nadeau V., Gagnon K., Mazoyer F. (2020). Identification of Long Noncoding RNA H19 as a New Biomarker and Therapeutic Target in Right Ventricular Failure in Pulmonary Arterial Hypertension. Circulation.

[13] Qin S., Predescu D., Carman B., Patel P., Chen J., Kim M., Lahm T., Geraci M., Predescu S.A. (2021). Up-Regulation of the Long Noncoding RNA X-Inactive–Specific Transcript and the Sex Bias in Pulmonary Arterial Hypertension. Am. J. Pathol..

[14] Zheng D., Hou Y., Li Y., Bian Y., Khan M., Li F., Huang L., Qiao C. (2020). Long Non-coding RNA Gas5 Is Associated With Preeclampsia and Regulates Biological Behaviors of Trophoblast via MicroRNA-21. Front. Genet..

[15] Jin L., Lin X., Yang L., Fan X., Wang W., Li S., Li J., Liu X., Bao M., Cui X. (2018). AK098656, a Novel Vascular Smooth Muscle Cell–Dominant Long Noncoding RNA, Promotes Hypertension. Hypertension.

[16] Peng W., Cao H., Liu K., Guo C., Sun Y., Qi H., Liu Z., Xie Y., Liu X., Li B. (2020). Identification of lncRNA-NR_104160 as a biomarker and construction of a lncRNA-related ceRNA network for essential hypertension. Am. J. Transl. Res..

[17] Yin L., Yao J., Deng G., Wang X., Cai W., Shen J. (2020). Identification of candidate lncRNAs and circRNAs regulating WNT3/β-catenin signaling in essential hypertension. Aging.

[18] Martinez-Arroyo O., Ortega A., Redon J., Cortes R. (2021). Therapeutic Potential of Extracellular Vesicles in Hypertension-Associated Kidney Disease. Hypertension.

[19] Tan P.P.S., Hall D., Chilian W.M., Chia Y.C., Zain S.M., Lim H.M., Kumar D.N., Ching S.M., Low T.Y., Noh M.F.M. (2021). Exosomal microRNAs in the development of essential hypertension and its potential as biomarkers. Am. J. Physiol. Circ. Physiol..

[20] Li Y., Meng Y., Liu Y., Van Wijnen A.J., Eirin A., Lerman L.O. (2021). Differentially Expressed Functional LncRNAs in Human Subjects With Metabolic Syndrome Reflect a Competing Endogenous RNA Network in Circulating Extracellular Vesicles. Front. Mol. Biosci..

[21] Perez-Hernandez J., Riffo-Campos A.L., Ortega A., Martinez-Arroyo O., Perez-Gil D., Olivares D., Solaz E., Martinez F., Martínez-Hervás S., Chaves F.J. (2021). Urinary- and Plasma-Derived Exosomes Reveal a Distinct MicroRNA Signature Associated With Albuminuria in Hypertension. Hypertension.

[22] Michalik K.M., You X., Manavski Y., Doddaballapur A., Zörnig M., Braun T., John D., Ponomareva Y., Chen W., Uchida S. (2014). Long Noncoding RNA MALAT1 Regulates Endothelial Cell Function and Vessel Growth. Circ. Res..

[23] Wang Y.-N.-Z., Shan K., Yao M.-D., Yao J., Wang J.-J., Li X., Liu B., Zhang Y.-Y., Ji Y., Jiang Q. (2016). Long Noncoding RNA-GAS5. Hypertension.

[24] Bell R.D., Long X., Lin M., Bergmann J.H., Nanda V., Cowan S.L., Zhou Q., Han Y., Spector D.L., Zheng D. (2014). Identification and Initial Functional Characterization of a Human Vascular Cell–Enriched Long Noncoding RNA. Arter. Thromb. Vasc. Biol..

[25] Villarroya-Beltri C., Gutierrez-Vazquez C., Sanchez-Cabo F., Pérez-Hernández D., Vázquez J., Martin-Cofreces N., Martinez-Herrera D.J., Pascual-Montano A., Mittelbrunn M., Sánchez-Madrid F. (2013). Sumoylated hnRNPA2B1 controls the sorting of miRNAs into exosomes through binding to specific motifs. Nat. Commun..

[26] Vickers K.C., Palmisano B.T., Shoucri B.M., Shamburek R.D., Remaley A.T. (2011). MicroRNAs are transported in plasma and delivered to recipient cells by high-density lipoproteins. Nat. Cell Biol..

[27] Yoon J.-H., Abdelmohsen K., Gorospe M. (2013). Posttranscriptional Gene Regulation by Long Noncoding RNA. J. Mol. Biol..

[28] Taverna S.D., Li H., Ruthenburg A., Allis C.D., Patel D.J. (2007). How chromatin-binding modules interpret histone modifications: Lessons from professional pocket pickers. Nat. Struct. Mol. Biol..

[29] Zhou Z., Li X., Liu Z., Huang L., Yao Y., Li L., Chen J., Zhang R., Zhou J., Wang L. (2020). A Bromodomain-Containing Protein 4 (BRD4) Inhibitor Suppresses Angiogenesis by Regulating AP-1 Expression. Front. Pharmacol..

[30] Du Y., Topp C., Dawe R.K. (2010). DNA Binding of Centromere Protein C (CENPC) Is Stabilized by Single-Stranded RNA. PLoS Genet..

[31] Fang Y., Fullwood M.J. (2016). Roles, Functions, and Mechanisms of Long Non-coding RNAs in Cancer. Genom. Proteom. Bioinform..

[32] Hsin J.-P., Manley J.L. (2012). The RNA polymerase II CTD coordinates transcription and RNA processing. Genes Dev..

[33] Ribeiro D., Zanzoni A., Cipriano A., Ponti R.D., Spinelli L., Ballarino M., Bozzoni I., Tartaglia G.G., Brun C. (2017). Protein complex scaffolding predicted as a prevalent function of long non-coding RNAs. Nucleic Acids Res..

[34] Shiraishi-Yamaguchi Y., Furuichi T. (2007). The Homer family proteins. Genome Biol..

[35] Pelava A., Schneider C., Watkins N.J. (2016). The importance of ribosome production, and the 5S RNP–MDM2 pathway, in health and disease. Biochem. Soc. Trans..

[36] Saito R., Rocanin-Arjo A., You Y.-H., Darshi M., Van Espen B., Miyamoto S., Pham J., Pu M., Romoli S., Natarajan L. (2016). Systems biology analysis reveals role of MDM2 in diabetic nephropathy. JCI Insight.

[37] Wang W., Zhu N., Yan T., Shi Y.-N., Chen J., Zhang C.-J., Xie X.-J., Liao D.-F., Qin L. (2020). The crosstalk: Exosomes and lipid metabolism. Cell Commun. Signal..

[38] Grevengoed T.J., Klett E.L., Coleman R.A. (2014). Acyl-CoA Metabolism and Partitioning. Annu. Rev. Nutr..

[39] Kotani A., Ito M., Kudo K. (2021). Non-coding RNAs and lipids mediate the function of extracellular vesicles in cancer cross-talk. Semin. Cancer Biol..

[40] Klebanov S.E., Bochina I.M., Berman A.L. (1991). A method for determining the classification of a protein in a given family at a low level of similarity. Zh. Evol. Biokhim. Fiziol..

[41] Dilmaghnai N.A., Shoorei H., Sharifi G., Mohaqiq M., Majidpoor J., Dinger M.E., Taheri M., Ghafouri-Fard S. (2021). Non-coding RNAs modulate function of extracellular matrix proteins. Biomed. Pharmacother..

[42] D’Angelo E., Agostini M. (2018). Long non-coding RNA and extracellular matrix: The hidden players in cancer-stroma cross-talk. Non-Coding RNA Res..

[43] Qiu Y., Li P., Zhang Z., Wu M. (2021). Insights Into Exosomal Non-Coding RNAs Sorting Mechanism and Clinical Application. Front. Oncol..

[44] Hu H., Ling B., Shi Y., Wu H., Zhu B., Meng Y., Zhang G.-M. (2021). Plasma Exosome-Derived SENP1 May Be a Potential Prognostic Predictor for Melanoma. Front. Oncol..

[45] Mancia G., Fagard R., Narkiewicz K., Redon J., Zanchetti A., Böhm M., Christiaens T., Cífková R., De Backer G., Dominiczak A. (2013). 2013 ESH/ESC Practice Guidelines for the Management of Arterial Hypertension. Blood Press..

[46] Olivares D., Perez-Hernandez J., Perez-Gil D., Chaves F.J., Redon J., Cortes R. (2020). Optimization of small RNA library preparation protocol from human urinary exosomes. J. Transl. Med..

[47] Wingett S.W., Andrews S. (2018). FastQ Screen: A tool for multi-genome mapping and quality control. F1000Research.

[48] Dobin A., Davis C.A., Schlesinger F., Drenkow J., Zaleski C., Jha S., Batut P., Chaisson M., Gingeras T.R. (2013). STAR: Ultrafast universal RNA-seq aligner. Bioinformatics.

[49] Li H., Handsaker B., Wysoker A., Fennell T., Ruan J., Homer N., Marth G., Abecasis G., Durbin R. (2009). 1000 Genome Project Data Processing Subgroup. The Sequence Alignment/Map format and SAMtools. Bioinformatics.

[50] Frankish A., Diekhans M., Ferreira A.-M., Johnson R., Jungreis I., Loveland J., Mudge J.M., Sisu C., Wright J., Armstrong J. (2019). GENCODE reference annotation for the human and mouse genomes. Nucleic Acids Res..

[51] Wang J., Zhang P., Lu Y., Li Y., Zheng Y., Kan Y., Chen R., He S. (2018). piRBase: A comprehensive database of piRNA sequences. Nucleic Acids Res..

[52] Robinson M.D., McCarthy D.J., Smyth G.K. (2010). EdgeR: A Bioconductor package for differential expression analysis of digital gene expression data. Bioinformatics.

[53] Fukunaga T., Iwakiri J., Ono Y., Hamada M. (2019). LncRRIsearch: A Web Server for lncRNA-RNA Interaction Prediction Integrated With Tissue-Specific Expression and Subcellular Localization Data. Front. Genet..

[54] Liao Y., Wang J., Jaehnig E.J., Shi Z., Zhang B. (2019). WebGestalt 2019: Gene set analysis toolkit with revamped UIs and APIs. Nucleic Acids Res..

[55] Szklarczyk D., Gable A.L., Lyon D., Junge A., Wyder S., Huerta-Cepas J., Simonovic M., Doncheva N.T., Morris J.H., Bork P. (2019). STRING v11: Protein–protein association networks with increased coverage, supporting functional discovery in genome-wide experimental datasets. Nucleic Acids Res..

[56] Shannon P., Markiel A., Ozier O., Baliga N.S., Wang J.T., Ramage D., Amin N., Schwikowski B., Ideker T. (2003). Cytoscape: A software environment for integrated models of Biomolecular Interaction Networks. Genome Res..

