# Supplementary material for: Biofluid Specificity of Long Non-Coding RNA Profile in Hypertension: Relevance of Exosomal Fraction
Source: Int J Mol Sci. 2022 May 6;23(9):5199. doi: 10.3390/ijms23095199 (PMC9101961; doi:10.3390/ijms23095199)
Supplement: Supplementary file 1 [file ijms-23-05199-s001.zip › ijms-1651158-supplementary.pdf]

Supplemental Material

Biofluid specificity of long non-coding RNA profile in hypertension: relevance of exosomal fraction

Angela L. Riffo-Campos [1,2,†], Javier Perez-Hernandez [3,†,5], Olga Martinez-Arroyo [3,†], Ana Ortega [3,*], Ana Flores-Chova [3], Josep Redon [3,5,6,‡] and Raquel Cortes [3,‡,*]

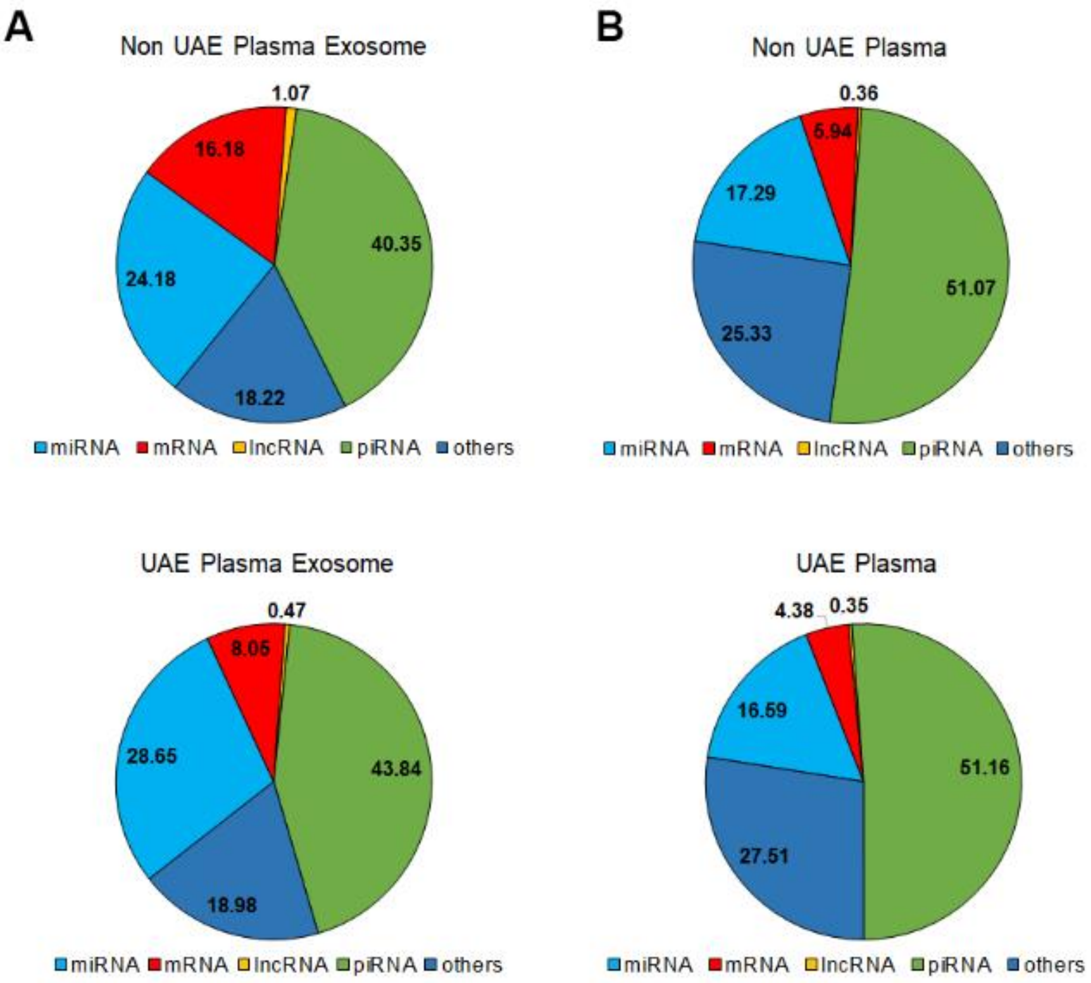

Figure S1. Proportion of non- coding RNAs according to their biofluid of origin and urinary albumin excretion presence. The proportion of RNA biotypes present in each of the two biological biofluids, plasma exosome and plasma, comparing patient's groups with and without UAE. lncRNA: long non-coding RNA; miRNA: microRNA; mRNA: messenger RNA; piRNA: PIWI-interacting RNA.

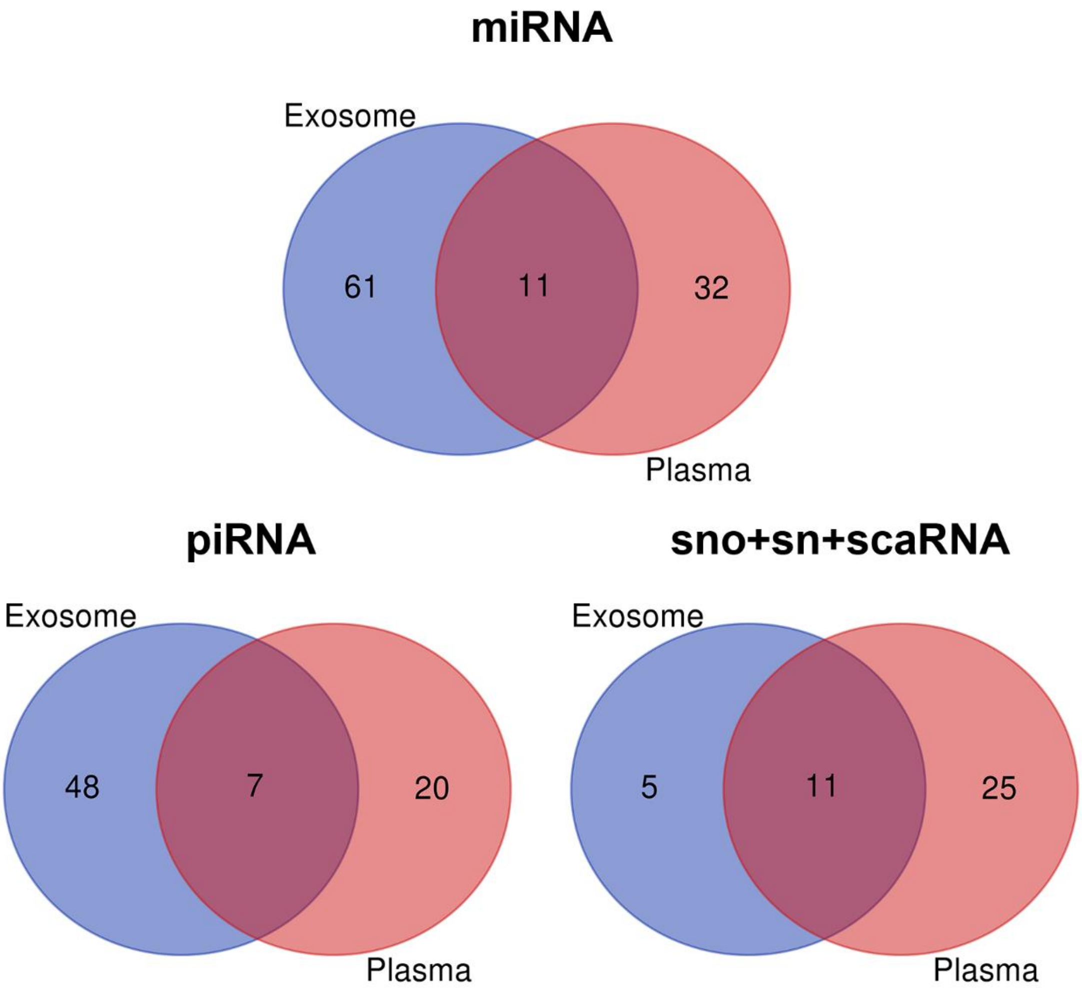

Figure S2. Differentially expressed non- coding RNAs according to their biofluid of origin. Overlapping of the differentially expressed miRNAs, piRNAs and sno+sn+scaRNA biotypes between plasma and exosomes.

Supplemental Table S1. List of top 25 differentially expressed lncRNA in plasma exosomes and plasma

Plasma Exosome				Plasma				
	SYMBOL	Log_2 FC	PValue		SYMBOL	logFC	logCPM	PValue
ENSG00000231290	APCDD1L-DT	5,88706308	2,03E-08	ENSG00000082929	LINC01587	-4,61448542	3,20863711	7,43E-05
ENSG00000223786	LOC101928516	4,87304012	3,11E-08	ENSG00000257636	G2E3-AS1	2,68357952	6,77695914	0,00020084
ENSG00000247199	LOC102546294	4,44181785	8,30E-08	ENSG00000177738	LOC648987	2,41084139	2,25359801	0,00046525
ENSG00000231607	DLEU2	3,4557984	1,64E-07	ENSG00000203875	SNHG5	2,39729814	6,40074701	0,00123688
ENSG00000185332	TMEM105	4,15655187	6,33E-07	ENSG00000224074	LINC00691	-2,9891942	2,802725	0,00134138
ENSG00000270641	TSIX	3,21630197	9,54E-06	ENSG00000267057	LINC01905	-3,05571791	3,07539723	0,00222363
ENSG00000216863	LY86-AS1	-3,60795138	1,64E-05	ENSG00000235760	HCG2040054	1,43438378	1,96774707	0,00230579
ENSG00000246090	LOC100507053	4,35078316	1,83E-05	ENSG00000225649	LOC100506474	-2,73140793	3,63193828	0,00278631
ENSG00000237879	LINC00398	-3,57937995	1,90E-05	ENSG00000282164	PEG13	-2,38124758	2,84795013	0,00326194
ENSG00000262772	LINC01977	-4,4398819	2,18E-05	ENSG00000245937	LINC01184	1,55668315	2,15248025	0,00371231
ENSG00000241696	LINC02053	-3,49960281	2,31E-05	ENSG00000261455	LINC01003	-2,41038825	1,83281703	0,0038277
ENSG00000186235	LINC02610	-3,20853542	2,48E-05	ENSG00000224743	TEX26-AS1	-2,10703507	5,17135437	0,0045258
ENSG00000281732	LOC284933	3,50476947	2,55E-05	ENSG00000281649	EBLN3P	-1,60174284	1,42535821	0,00612789
ENSG00000263069	LOC100294362	-3,27905099	3,55E-05	ENSG00000251576	LINC01267	1,98482107	1,95760394	0,00728475

ENSG00000267289	LOC100996288	-4,0014274	3,70E-05	ENSG00000241684	ADAMTS9-AS2	-1,6774242	3,16348326	0,00785206
ENSG00000253230	LINC00599	-3,15684096	6,10E-05	ENSG00000223764	LINC02593	1,65375336	1,64723791	0,00993578
ENSG00000196756	SNHG17	-3,09032887	6,65E-05	ENSG00000212766	EWSAT1	-1,89146711	2,84414508	0,01011951
ENSG00000226674	TEX41	2,65225019	7,38E-05	ENSG00000183250	LINC01547	1,64843315	2,76625381	0,0111592
ENSG00000233223	LOC100996842	-3,63591811	0,00010701	ENSG00000174365	SNHG11	1,79846042	2,13198997	0,01171103
ENSG00000267201	LINC01775	-4,47146662	0,00010827	ENSG00000237187	NR2F1-AS1	-1,331982	3,02537356	0,01222882
ENSG00000228794	LINC01128	3,06986857	0,00011905	ENSG00000268460	LOC93429	-1,4838163	1,49081222	0,01228452
ENSG00000261504	LINC01686	-3,10480548	0,00012288	ENSG00000233593	LINC02609	1,60615613	6,02725905	0,01268583
ENSG00000205181	LINC00654	3,38920978	0,00013002	ENSG00000234912	SNHG20	-1,36606269	2,1052033	0,0150422
ENSG00000256073	URB1-AS1	3,19631016	0,00013672	ENSG00000251173	UCHL1-AS1	1,21588416	2,31797867	0,01650519
ENSG00000224424	PRKAR2A-AS1	-3,4343828	0,00014186	ENSG00000231419	LINC00689	-1,89184633	3,13699976	0,01712979

Table S2. List of the top 25 lncRNA targets in plasma and plasma exosomes.

Plasma lncRNA targets		Plasma exosome targets	
Gene	Type	Gene	Type
ABC13-47488600E17.1	Protein Coding	AATBC	Protein Coding
**ABI2**	Protein Coding	**ABI2**	Protein Coding
**AC006548.28**	lncRNA	**AC006548.28**	lncRNA
AGO3	Protein Coding	ACOT11	Protein Coding
AIRN	lncRNA	ADH1A	Protein Coding
AP006621.9	lncRNA	ADH1C	Protein Coding
ATP5A1	Protein Coding	ANKRD34A	Protein Coding
**ATXN3**	Protein Coding	AP002962.1	lncRNA
**AURKC**	Protein Coding	AP006621.9	lncRNA
**B3GALNT2**	Protein Coding	**ATXN3**	Protein Coding
**BCAM**	Protein Coding	**AURKC**	Protein Coding
BRD4	Protein Coding	**B3GALNT2**	Protein Coding
C10orf91	Protein Coding	**BCAM**	Protein Coding
**C9orf152**	Protein Coding	C20orf96	Protein Coding
**CCDC85C**	Protein Coding	**C9orf152**	Protein Coding
CENPB	Protein Coding	CADM2-AS2	lncRNA
CTA-212A2.3	lncRNA	**CCDC85C**	Protein Coding
CTA-228A9.4	lncRNA	CNKSR3	Protein Coding
**CTA-992D9.11**	lncRNA	CTA-280A3.2	lncRNA
**CTB-176F20.3**	lncRNA	**CTA-992D9.11**	lncRNA
CTD-2144E22.9	lncRNA	**CTB-176F20.3**	lncRNA
DCAF8L2	Protein Coding	CTC-360G5.9	lncRNA
**DLK1**	Protein Coding	CTD-2006C1.2	lncRNA
**EMC10**	Protein Coding	DBET	Protein Coding
**ERICH6**	Protein Coding	DHODH	Protein Coding
FAM109A	Protein Coding	**DLK1**	Protein Coding
**FAM122B**	Protein Coding	DONSON	Protein Coding
**FAM98B**	Protein Coding	**EMC10**	Protein Coding
FLG	Protein Coding	**ERICH6**	Protein Coding
**FLJ35934**	lncRNA	**FAM122B**	Protein Coding
**GABPB1-AS1**	lncRNA	**FAM98B**	Protein Coding
GAD2	Protein Coding	FGD3	Protein Coding
**GAS6-AS1**	lncRNA	**FLJ35934**	lncRNA
**GNB5**	Protein Coding	FN3K	Protein Coding
**GOLGA6L1**	Protein Coding	**GABPB1-AS1**	lncRNA
**GOLGA6L2**	Protein Coding	**GAS6-AS1**	lncRNA
**GOLGA6L22**	Protein Coding	**GNB5**	Protein Coding
**GOLGA6L6**	Protein Coding	**GOLGA6L1**	Protein Coding
**GOLGA6L7P**	Protein Coding	**GOLGA6L2**	Protein Coding
**GS1-519E5.1**	lncRNA	**GOLGA6L22**	Protein Coding
**HELLPAR**	lncRNA	**GOLGA6L6**	Protein Coding
IGF2	Protein Coding	**GOLGA6L7P**	Protein Coding

IGFN1	Protein Coding	**GS1-519E5.1**	lncRNA
KCNJ6	Protein Coding	**HELLPAR**	lncRNA
**KCNQ1OT1**	lncRNA	**KCNQ1OT1**	lncRNA
KMT2D	Protein Coding	KRTAP4-16	Protein Coding
KRT9	Protein Coding	**LA16c-358B7.4**	lncRNA
KSR2	Protein Coding	LINC00482	lncRNA
**LA16c-358B7.4**	lncRNA	**LINC00940**	lncRNA
**LINC00940**	lncRNA	lnc-TMEM105	lncRNA
LL0XNC01-16G2.1	lncRNA	LOC112268186	lncRNA
LL22NC03-86G7.1	lncRNA	MGAT4C	Protein Coding
LRRK1	Protein Coding	**MIR6820**	miRNA
MDM2	Protein Coding	**MLYCD**	Protein Coding
**MIR6820**	miRNA	MRRF	Protein Coding
**MLYCD**	Protein Coding	**MUC12**	Protein Coding
MRPL42	Protein Coding	MUC3A	Protein Coding
**MUC12**	Protein Coding	**MUC4**	Protein Coding
MUC16	Protein Coding	**NBPF19**	Protein Coding
MUC2	Protein Coding	**NBPF20**	Protein Coding
**MUC4**	Protein Coding	NEAT1	Protein Coding
MUC6	Protein Coding	NIM1K	Protein Coding
NBPF10	Protein Coding	NTRK3	Protein Coding
NBPF14	Protein Coding	ORAI2	Protein Coding
**NBPF19**	Protein Coding	**PARP11**	Protein Coding
**NBPF20**	Protein Coding	**PARVG**	Protein Coding
NDUFA10	Protein Coding	PAX2	Protein Coding
**PARP11**	Protein Coding	**PDX1**	Protein Coding
**PARVG**	Protein Coding	**PLD4**	Protein Coding
**PDX1**	Protein Coding	PRKX	Protein Coding
PLAC4	lncRNA	PRR21	Protein Coding
PLBD2	Protein Coding	RF00017	Protein Coding
**PLD4**	Protein Coding	RNF213	Protein Coding
PNMA5	Protein Coding	RP11-156E8.1	lncRNA
POLR2A	Protein Coding	RP11-473M20.9	lncRNA
POU2AF1	Protein Coding	RP13-580B18.4	lncRNA
PPP1R18	Protein Coding	**RP3-323A16.1**	lncRNA
PRG4	Protein Coding	**RP5-1014D13.2**	lncRNA
PRR12	Protein Coding	**RPGR**	Protein Coding
PRR36	Protein Coding	**RPS6KA5**	Protein Coding
PRRC2	Protein Coding	SDK1	Protein Coding
RP11-186N15.3	lncRNA	SENP2	Protein Coding
RP11-338K13.1	lncRNA	SIX3	Protein Coding
RP11-573D15.8	lncRNA	**SLC35E3**	Protein Coding
RP11-640N11.2	lncRNA	SLC38A10	Protein Coding
RP11-95O2.5	lncRNA	**SNX8**	Protein Coding
**RP3-323A16.1**	lncRNA	SPN	Protein Coding

RP3-394A18.1	lncRNA	SSC5D	Protein Coding
**RP5-1014D13.2**	lncRNA	THEM5	Protein Coding
**RPGR**	Protein Coding	TMCC1-AS1	lncRNA
**RPS6KA5**	Protein Coding	TMEM105	lncRNA
SEC14L1	Protein Coding	**TNFRSF13B**	Protein Coding
SHANK1	Protein Coding	TPSD1	Protein Coding
**SLC35E3**	Protein Coding	**TSIX**	lncRNA
**SNX8**	Protein Coding	**TTC34**	Protein Coding
SOGA1	Protein Coding	**UCN3**	Protein Coding
SRCAP	Protein Coding	USB1	Protein Coding
**TNFRSF13B**	Protein Coding	**USP8**	Protein Coding
TRIM41	Protein Coding	WAC	Protein Coding
**TSIX**	lncRNA	**WDR78**	Protein Coding
**TTC34**	Protein Coding	**XX-C00717C00720L.1**	lncRNA
**UCN3**	Protein Coding	**YJEFN3**	Protein Coding
**USB1**	Protein Coding	ZNF154	Protein Coding
USP8	Protein Coding	ZNF471	Protein Coding
VGF	Protein Coding		
**WDR78**	Protein Coding		
**XX-C00717C00720L.1**	lncRNA		
**YJEFN3**	Protein Coding		
ZBTB47	Protein Coding		
ZNF316	Protein Coding		

Bold letter indicates common lncRNA targets for both biofluids. LncRNA: long non-coding RNA.

Supplemental Table S3. List of targets of the lncRNA network with high degree node

Symbol name	Biofluid	Degree	Betweenness Centrality	Closeness Centrality
RPGR	Common	46	0.08435476	0.50212766
RP11-573D15.8-018	Common	38	0.04162673	0.46732673
ABI2	Common	38	0.05081476	0.46548323
SNX8	Common	37	0.05589426	0.46732673
LOC100507053	Exosomes	34	0.10620332	0.40901213
FAM98B	Common	33	0.03399844	0.4469697
EBLN3P	Plasma	30	0.03979936	0.41330998
LINC01184	Plasma	30	0.03364524	0.41769912
CCDC85C	Common	28	0.02165491	0.43065693
EMC10	Common	28	0.02141953	0.42831216
LINC01686	Exosomes	28	0.03254247	0.41330998
UCHL1-AS1	Plasma	28	0.0571334	0.40759931
LINC00689	Plasma	28	0.02138605	0.4034188
LINC01128	Exosomes	27	0.05368149	0.42067736
LINC00654	Exosomes	27	0.01586564	0.4
LY86-AS1	Exosomes	27	0.02248016	0.40136054
RP3-323A16.1	Common	26	0.02924652	0.42293907
APCDD1L-DT	Exosomes	25	0.03225851	0.40410959
MIR7515HG	Plasma	25	0.04248146	0.4034188
SNHG11	Plasma	25	0.05302646	0.39202658
DLEU2	Exosomes	24	0.02791779	0.40480274
TEX26-AS1	Plasma	24	0.01588386	0.39597315
SNHG5	Plasma	24	0.04441488	0.40901213
GOLGA6L22	Common	23	0.01511363	0.41843972
LINC02609	Plasma	23	0.03601183	0.40204429
LOC100996842	Exosomes	22	0.0609892	0.3728278
KMT2D	Plasma	22	0.03210597	0.41549296
LOC648987	Plasma	21	0.0131542	0.3973064
MIR6820	Common	20	0.0147587	0.39530988
POLR2A	Common	20	0.02033848	0.40273038
LINC01775	Exosomes	20	0.01711326	0.38879736
GOLGA6L6	Common	19	0.00941249	0.39663866
TEX41	Exosomes	19	0.02423112	0.39663866
MDM2	Plasma	19	0.01425838	0.41114983
LOC100506474	Plasma	18	0.02344729	0.38064516
LRRK1	Plasma	18	0.02229349	0.40410959
G2E3-AS1	Plasma	18	0.01075088	0.38499184
PARVG	Common	17	0.01136836	0.37579618
LOC100294362	Exosomes	17	0.01274382	0.37820513
LINC01905	Plasma	17	0.01338578	0.37106918
PDX1	Common	16	0.01755364	0.40549828
HELLPAR	Common	16	0.02093184	0.38499184

SLC35E3	Common	16	0.00819701	0.40273038
USP8	Common	16	0.00870224	0.37942122
NTRK3	Exosomes	16	0.01896487	0.39797639
SNHG20	Plasma	16	0.00582042	0.36760125
GNB5	Common	15	0.02287953	0.38815789
AP006621.9	Common	15	0.00380683	0.36307692
LINC02610	Exosomes	15	0.01431498	0.3776
LINC00691	Plasma	15	0.00531352	0.39008264
EWSAT1	Plasma	15	0.00355016	0.3728278
SHANK1	Plasma	15	0.01982766	0.40972222
GOLGA6L1	Common	14	0.00593183	0.37942122
TSIX	Common	14	0.00776549	0.40480274
NR2F1-AS1	Plasma	14	0.02778832	0.37639553
AURKC	Common	13	0.01802841	0.4083045
GOLGA6L2	Common	13	0.00746063	0.36532508
LINC00940	Common	13	0.01000249	0.38499184
USB1	Common	13	0.01301608	0.39464883
ATXN3	Common	13	0.00923119	0.38003221
GAD2	Plasma	13	0.00891568	0.36419753
LOC93429	Plasma	13	0.00328406	0.37341772
MUC4	Common	12	0.00744632	0.36419753
RPS6KA5	Common	12	0.00646665	0.38187702
LINC01587	Plasma	12	0.01110853	0.36702955
BRD4	Plasma	12	0.00488659	0.40549828
WDR78	Common	11	0.008144	0.38879736
LA16c-358B7.4	Common	11	0.01033898	0.38562092
PRKX	Exosomes	11	0.01280836	0.34962963
LOC284933	Exosomes	11	0.00162451	0.3728278
MUC12	Common	11	0.00725362	0.35171386
WAC	Exosomes	11	0.00711146	0.37165354
ago-03	Common	11	0.00721278	0.38625205
IGF2	Plasma	11	0.0072442	0.40759931
FLJ35934	Common	10	0.007326	0.3728278
LOC102546294	Exosomes	10	0.00137937	0.35757576
CTA-992D9.11	Common	10	0.0029267	0.3480826
B3GALNT2	Common	10	0.00408261	0.33475177
CTA-228A9.4	Common	10	0.00206594	0.3480826
DLK1	Common	10	0.00581081	0.3776
GS1-519E5.1	Common	10	0.00281145	0.35223881
LINC01977	Exosomes	10	0.03045004	0.3425254
LOC100996288	Exosomes	10	0.0010608	0.37106918
MUC5AC	Common	10	0.00583	0.34756996
MUC6	Common	10	0.00527589	0.34962963
URB1-AS1	Exosomes	10	9,09E+00	0.36030534
LINC00398	Exosomes	10	8.39E-4	0.36196319

PAX2	Exosomes	10	0.00556774	0.3597561
SNHG17	Exosomes	10	0.00500903	0.36476043
PEG13	Plasma	10	0.00114341	0.3597561
ADAMTS9-AS2	Plasma	10	9,68E+00	0.36030534
KSR2	Plasma	10	0.00548123	0.38815789
ATP5A1	Plasma	10	0.00380878	0.34756996
HCG2040054	Plasma	10	0.00927308	0.35171386
LINC01267	Plasma	10	0.00284009	0.36307692
LINC02593	Plasma	10	0.00141883	0.37699681
LINC01547	Plasma	10	7,72E+00	0.36817473

Selected targets with a degree greater than 10.

Supplemental Table S4. List of the most relevant pathways identified by GO and KEGG analysis with their involved transcripts

Plasma exosome			Plasma		
**Pathway**	**GO Terms**	**Transcript**	**Pathway**	**GO Terms**	**Transcript**
Fatty acid metabolism	GO:0047617 acyl-CoA hydrolase activity	THEM5;ACOT11	histone or chromatin remodeling	GO:0042393 histone binding	BRD4;SRCAP;KMT2D
	GO:0016289 CoA hydrolase activity	THEM5;ACOT11		GO:0019237 centromeric DNA binding	CENPB
	GO:0016790 thiolester hydrolase activity	THEM5;ACOT11		GO:0003696 satellite DNA binding	CENPB
	GO:0036042 long-chain fatty acyl-CoA binding	THEM5;ACOT11		GO:0042800 histone methyltransferase activity	KMT2D
ECM binding, laminin binding	GO:0030197 extracellular matrix binding	BCAM;SSC5D	RNA polymerase	GO:0099122 RNA polymerase II C-terminal domain binding	BRD4
	GO:0043236 laminin binding	BCAM;SSC5D		GO:0001055 RNA polymerase II activity	POLR2A
Sorting protein and ncRNAs	GO:0070138 ubiquitin-like protein-specific isopeptidase activity	SENP1; SENP2	scaffold protein binding	GO:0097110 scaffold protein binding	SHANK1;MDM2
	GO:0070140 SUMO-specific isopeptidase activity	SENP1; SENP2		GO:0008097 5S rRNA binding	MDM2
				GO:0002039 p53 binding	SHANK1;MDM2
